# The Great Concert and Two Important Speeches

**Published:** 1917-05-05

**Authors:** 


### THE ROYAL COLLEGE OF BRITISH NURSING.

### The Great Concert and Two Important Speeches.

A concert in honour of British Hospital Nurses was given at the Queen's Hall on Wednesday, May 2, 1917, under the gracious patronage of Her Majesty Queen Alexandra, and of Her Royal Highness Princess Christian of Schleswig-Holstein, organised by Mr. Victor Beigel under the auspices of the College of Nursing.

This concert should make Mr. Victor Beigel famous as a master of musical organisation, for it began to time and ended at five o'clock to the minute. It was splendid, for there were several well-earned encores which took extra time. Mr. Mark Hambourg, whose piano-playing and solo were magnificent, carried the audience completely away and united them in enthusiastic acknowledgments. Miss Louise Dale sang exquisitely two well-chosen songs. Her execution and voice were perfect, and nothing better has been heard in Queen's Hall for a long time. Miss Olga Nethersole gave three short recitations, which markedly moved the audience, and her encore "England" brought down the house. Miss Beatrice Harrison's 'cello solos were charming; the first one, "The Lament," by Victor Beigel, its first performance, is likely to be heard often and to become, deservedly, a recognised favourite. Mdlle. Zoe Rosowsky sang an aria from "Madama Butterfly" and a song, "Le Temps des Lilas," by Chausson. Both were rendered with a reserve and force which gave them distinction. M. Yves Tinayre sang two French songs, "Chanson Triste" and "Le Roi d'Ys," with a native charm which would have enthused a French audience, as it did many of those present in Queen's Hall. Mr. Madoc Davies' songs, "In Summer-time on Bredon" and "Speed the Plough," were excellent in their way. We hope to have the pleasure of hearing them both again quickly. M. Albert Fransella's flute obbligato was delicately rendered, and the violin soli, "Irish Air" and "Spanish Dance" (Sarasate), by Miss Ivy Angove, were played with a mastery of her instrument and an execution and skill which mark her out for fame of the highest. Madame Alexia Bassian, in the aria "Mon cœur s'ouvre à ta voix" ("Samson et Delila"), sang with a charm which made it an inspiration to listen to her. The Westminster Singers, as usual, were great in two quartettes, "The Charge of the Bargain Brigade," a skit on women's shopping, and "The Patriotic Fowl," a new catch appropriate to the war, which highly entertained the audience. "Land of Hope and Glory," by Elgar, was down on the programme "to be sung by the audience." The organist did not help the latter by playing, at any rate, some bars of the air, so that the first eight lines were not sung, for few present seemed to know the music. The chorus, however, was taken up with great energy, and was sung lustily by the whole of those present with good effect. Few, if any, concerts were better worth hearing. Indeed, the success of Mr. Victor Beigel's generous and kindly enterprise was so complete that this concert will long and pleasurably linger in the memory of many who spent a delightful afternoon at Queen's Hall.

### Speech by the Hon. Arthur Stanley, M.P.

Your Majesty, Your Royal Highness, Ladies and Gentlemen,—I see on the programme—the front page of the programme—that I am put down for an address. Well, I can assure you, both for your own satisfaction and for mine, that I do not contemplate any such terrible thing. My real reason for this intrusion is to voice what I am sure you all feel—an expression of our gratitude to Mr. Victor Beigel and to those who have got up the concert this afternoon. (Applause.) I had the pleasure of meeting Mr. Victor Beigel some little time ago, and he then told me of his thought of this concert. In connection with the Red Cross, I have heard of very many kindly actions and kindly thoughts, but I think this may be ranked as one of the kindest. Mr. Beigel has got up for the Red Cross nearly three hundred concerts in the various hospitals under our control. In all those concerts he has always seen a fair attendance of nurses, but he has noticed that the nurses themselves have always been in attendance on the patients, and he has felt that, instead of giving their full attention to the concert, and deriving the greatest possible pleasure from it, they were really giving a great deal more of their attention to the patients than to the concert. Well, he thought that he would like to give a concert to which the nurses should be asked without the patients, in order that the nurses might have the full enjoyment for themselves and for nobody else. (Applause.)

Now, when Mr. Beigel told me of that idea, I am sorry to have to confess that I saw in it a chance of doing what I wanted to do—namely, to ensure that the College of Nursing should make its first bow in connection with an act of real kindness towards the nurses. (Applause.) Of course, with a large audience of nurses like this, and in the presence of Her Gracious Majesty, who has ever been the kindest and best of friends to nurses and the nursing profession—(loud applause)—it is very tempting to me to make a speech to you about the College of Nursing. But in your interests, and out of kindness to you, I shall refrain. (Laughter.) I have chosen what I think you will all agree is the better part, and instead of making a speech about the College of Nursing I arranged that a leaflet on the subject should be placed in every chair. I feel very strongly on the subject of the necessity for a great Central Association for the nursing profession of this country. (Applause.) We have many difficulties yet to overcome, but I hope and believe that we are on the right line. (Renewed applause.) There is another association existing, as all nurses know, with very much the same end as ours—the Royal British Nurses' Association. We approached them with a view to seeing whether two Societies such as theirs and ours, having much the same aims and objects, could not join hands and work as one. I am happy to say that we were met by the Royal British Nurses' Association in the most friendly manner. Both Associations have unanimously voted in favour of the amalgamation; the agreement is now before the Privy Council; and when it goes through I hope we shall emerge as the Royal British College of Nursing, with very great benefit to ourselves and our cause, and having as our President Her Royal Highness Princess Christian. (Applause.) For thirty years or more Her Royal Highness has ever been supporting and standing up for the cause of the nurses, and I feel that if we have her Royal approval, we must indeed be on the right path. (Applause.)

### The Nation's Fund for Nurses.

Now there is one other leaflet that those of you who are observant may also have noticed on your seats, and that is the leaflet about the Nation's Fund for Nurses. Well, I will not make a speech about that either, but I

do commend that to your very earnest consideration. If we are going to have an effective central organisation, we must have money. If we are going to have money, unless it is going to come out of the pockets of the nurses themselves, which I very much doubt—(laughter)—we have got to get it out of somebody else's pocket. Now we have not started this Fund with the usual flourish of trumpets. We have let it steal quietly—stealing is a good word in this connection—(laughter)—we have let it steal quietly and unobtrusively into the world. And there is a reason for that. We do not want in this movement to start forward with great names or with large sums from distinguished people. What we want to do is that this Fund shall be a real tribute of gratitude from those who have had so much benefit themselves from the ministrations of the nurses. (Applause.) There are a very few of us who go through life without owing a debt of gratitude to the nurses, and at this moment, when literally hundreds of thousands of the brave men who are fighting for their country are being helped in their suffering and in their illness by the nurses, surely this is the right moment to ask the great British public to show its gratitude. (Applause.) I think one of the proudest inscriptions on any of the numerous statues in London is one in Trafalgar Square to one of our great Generals, where it says that the statue was erected by numerous contributions, and that among the most numerous subscribers were the private soldiers themselves. (Applause.) Well, if this Fund for Nurses is approached in the same spirit, then I feel perfectly certain that it will be a magnificent success, and that we shall have been instrumental, in these strenuous times, in doing something towards helping and furthering the noblest profession in the world and the most deserving of all women—the magnificent British Nurses of the British Empire. (Applause.)

Now, with that slight digression, I return to the object with which I appear on the stage. I shall move a very hearty vote of thanks to Mr. Victor Beigel and his friends; I will ask that it may be seconded—much better than I can do it myself—by Miss Compton, and Miss Compton will then have the very great pleasure of putting it to you for your approval. (Applause.)

### Speech by Miss Compton.

Your Majesty, Your Royal Highness, Ladies and Gentlemen,—I have been asked to add a few words in support of the proposed idea of a National Nursing College. In speaking to you I am representing the British Women's Hospital Committee, and I fancy some of our work may be known to you. It was recently suggested to us that this scheme of a National Nursing College was in the nature of a debt that the nation owed to its nurses, and it was suggested to our Committee that they would find congenial scope for their energies in trying to assist the movement. "Well," I said, "if the nurses of Great Britain want a College, and ask for a College, we will do our level best to help them to get it." (Applause.)

### The Professional and the Amateur.

There was one point that caught my eye in the short circular. It seemed to imply that this proposed College would fix a strong dividing-line between the trained professional nurse and the amateur. Amateurs are like the poor—they are always with us. (Laughter.) Many of them are well-intentioned; but it is sometimes difficult to say more for them. I am a member of the dramatic profession, which also has its amateur camp-followers. Our attitude towards them is tolerant rather than enthusiastic. (Laughter.) Just before the war, I heard of a young man who had done a great deal as an amateur, and he made up his mind to become a professional. And then—nothing happened. (Laughter.) A friend said to him, "I say, old chap, aren't you going on the stage?" He said, "Oh! yes; that's all right! I have sent my photograph to all the London managers, and now it's up to them!" (Renewed laughter.)

### A Heart in Need of Splints.

These are topsy-turvy times, and one's brain is in a whirl. I have been trying to keep up with the constant changes in the food restrictions—(laughter)—but it is no good; I cannot do it. What I seem to have found is this—that while we are waiting for peace, the most patriotic thing we can do is to quarrel with our bread and butter. (Renewed laughter.) Be that as it may, I do hope that after the war our poor old battered world will be allowed a short period of rest and convalescence before it begins to put its many houses in order. Speaking of convalescence reminds me of a picture I saw the other day. One of our dear boys in khaki was lying back in a comfortable deck-chair, while a most attractive hospital nurse was bending over him; and he was looking up in her face—shall I say gratefully? Underneath was written, "Not yet quite out of danger." I would not venture to question the sort of danger, but I am afraid that from the medical point of view the young soldier would have to undergo the painful operation of having his heart put into splints. (Laughter.)

### We Want, We Hope, and We Mean to Help You.

Well, now, as to this College question, I can only repeat that you can rely on our Committee to help you, and you can be sure of its sympathy. (Applause.) Why? Because we are women, and we are doubly proud of that fact when we remember what our nursing sisterhood has done, is doing, and will yet do. They have made heroism into a passion, and one seems to lose all sense of disproportion in the contemplation of this picture. There, at the bedside—the great emblem of human suffering—stands the nurse, through the day and through the night, embodying tenderness, arresting pain, challenging Death. This war has opened many avenues of work to women, and every woman who is worth her salt is proud of her own calling. I am a working woman, and I am very proud of mine. But there is not one of us who would not eagerly admit that among all the professions open to us that of the professional nurse is the finest, the greatest, the noblest of all. And we other women stand at ease, and salute the nurses of Great Britain, while they march on down the great, broad road of Mercy. That is why we want to help you, and that is why we hope to help you, and that is why we mean to help you if we can. (Applause.)

Now I want to second the resolution of thanks put forward by Mr. Stanley for the kindness shown by Mr. Victor Beigel and the artistes engaged in the brilliant programme that is being submitted to you. I now put it to the nurses for their approval. (Loud and continued applause.)

### Mr. Victor Beigel:

Your Majesty, Your Royal Highness, Ladies and Gentlemen, I am most grateful for the very kind words which have been said about me, and I should have limited myself, in this part of the performance, to acknowledging the kind words and hearty thanks which have been accorded; but I feel that it is only fair to tell you that from the very first, when I mentioned to the Council of the College of Nursing the idea of a concert in honour of British Hospital Nurses, as a small—a very small—token of our deep gratitude for what they have done in this war, and are still doing, the Council backed me up with the greatest enthusiasm in everything I asked them to do. They helped me and did everything that was in their power. Therefore I think it only just to express my hearty thanks to the Council of the College of Nursing. I will now detain you no longer. I only wish to express my hope that your pleasure in being here may be at all equal to our pleasure in welcoming you here to-day. (Applause.)

